# Editorial: Strategies for improving hypertension management

**DOI:** 10.3389/fmed.2022.1011957

**Published:** 2022-10-28

**Authors:** Komal Marwaha, Freny Vaghaiwalla Mody, Indranill Basu-Ray, Keith Curtis Norris

**Affiliations:** ^1^Department of Medical Education, Paul L Foster School of Medicine, Texas Tech University Health Science Center El Paso, El Paso, TX, United States; ^2^David Geffen School of Medicine, University of California, Los Angeles, Los Angeles, CA, United States; ^3^Memphis VA Medical Center, United States Department of Veterans Affairs Memphis, Memphis, TN, United States; ^4^Department of Medicine, David Geffen School of Medicine, University of California, Los Angeles, Los Angeles, CA, United States

**Keywords:** hypertension, intestinal barrier, financial incentives, masked hypertension, high-risk metabolic syndrome, blood group, nebivolol

Despite preventive strategies and major advances in pharmacological treatment, the prevalence of hypertension has been increasing (1). To curb this rise, there is a need to gather new evidence and critically re-evaluate preexisting evidence so strategies can be formulated to prevent hypertension and improve its management.

The pathogenesis of hypertension cannot be entirely elucidated by genetics and conventional risk factors (2). There is a need to identify and mitigate additional risk factors, evaluate the efficacy of anti-hypertensive drugs in different populations (3), and explore non-pharmacological treatment that could improve treatment adherence and long-term BP control (4). In this collection” Strategies for improved management of Hypertension,” Li C. et al. identified the role of impaired gut health in hypertension. They found elevated intestinal barrier serum markers diamine oxidase, lipopolysaccharide, and D-lactate in hypertensive patients compared to normotensives. The patients with longstanding hypertension history (≥20 years), poor control of diastolic blood pressure, and cardiac and renal complications had elevated diamine oxidase and lipopolysaccharide, while patients on multidrug therapy for hypertension had higher D-lactate levels. This study suggests the association of the intestinal barrier with hypertension.

In another study on the association of the ABO blood group with preeclampsia, Li T. et al. found that the AB blood group patients have an increased risk of preeclampsia (PE), and the A blood group is associated with early-onset PE. Clinicians could proactively manage pregnant women with these blood groups who have raised blood pressure and are potentially at high risk of developing PE.

Besides accumulating new evidence, the preexisting evidence applicability in different sets of populations could also be helpful. Ojji et al. found the selective beta-1 blocker nebivolol has significant antihypertensive efficacy and satisfactory tolerability in reducing both systolic blood pressure (SBP) and diastolic blood pressure (DBP) in Blacks with stage I hypertension residing in sub-Saharan Africa, indicating the potential use of nebivolol as monotherapy in grade 1 hypertension or as the fourth drug in multitherapy after maximum doses of a combination of long-acting calcium channel blockers, thiazide diuretics, and renin-angiotensin-aldosterone blockers have been used.

Sun et al. investigated the effect of an incremental rise in the risk of cardiovascular diseases (CVD) with increasing SBP in older adults above 65 years of age, having SBP between 90 and 129 mm Hg. The authors found a non-linear association between normal range SBP levels and all-cause and cardiovascular death. Also, when SBP <115 mmHg, the risk of mortality gradually increased with decreasing SBP, and when SBP ≥115 mmHg the risk of mortality gradually increased with SBP level. The findings suggest that SBP level 110–120 mmHg could be optimal, and the SBP level in the normal range might have a dose-response relationship with all-cause and cardiovascular mortality.

Yang et al. examined the relationship between SBP and in-hospital deaths in Type -A Aortic dissection (AAD) patients and found a non-linear correlation between admission SBP and in-hospital mortality of AAD patients when SBP was ≤120 mmHg. A SBP ≤ 120 mmHg negatively correlated with in-hospital mortality and a 10 mmHg rise in admission SBP was found to be associated with 12% lower odds of in-hospital mortality.

Fu et al. identified two sub-phenotypes in masked hypertension (MHT); the low-risk metabolic syndrome (MetS) and the high MetS (characterized by larger waist circumference, lower HDL-C, higher fasting blood glucose, and triglycerides, and prevalence of diabetes) sub-phenotype. The authors found a significant correlation between sub-phenotypes and clinical results and the sub-phenotypes have different metabolic characteristics and prognoses. For patients with MHT and high-risk MetS, antihypertensive therapy alone may be insufficient. In addition to antihypertensive treatment, the management of the metabolic disorder in MHT populations may be also considered in the future.

Visco et al. retrospectively analyzed the database of 177 essential hypertensives and found an independent positive correlation of serum uric acid (sUA) with left ventricular mass index (LVMi) which is a common complication of hypertension and increases the risk of cardiovascular morbidity. The results showed that the sUA above 5.6 mg/dl predicts larger LV sizes. The results suggest hyperuricemia is an early marker of increased left ventricular mass that can be used to identify a hypertensive population with target organ damage.

Meng and Xu stated that the ideal blood pressure for the treatment and management of Atrial Fibrillation (AF) is not clear. The eight major hypertension guidelines that were updated between 2016 and 2019 vary in the recommended target BP for preventing new-onset AF. The authors expressed that the ideal approach to assess the impact of hypertension on AF in large population-based cohort studies remains unclear because of the increased beat-to-beat BP variability in the presence of AF which may affect BP estimation and thus the assessment of the correlation between BP and AF. Also, the insufficient sensitivity in detecting sporadic arrhythmias (such as paroxysmal AF) by intermittent monitoring adds to the complexity of accuracy in answering the question. The authors suggested the use of a continuous rhythm monitoring strategy to overcome the limitations of intermittent monitoring in assessing the rate of incident AF and help evaluate the real effect of hypertension on AF.

Meier et al. evaluated whether the financial incentives are more effective than evidence-based educational feedback reports in the increasing proportion of patients with diabetes mellitus meeting specific quality indicators (QIs). The authors found that the intervention group achieved an improvement of 2% in the proportion of patients achieving the recommended blood pressure target levels, whereas the proportion of patients receiving annual HbA1c measurements remained stable. After the withdrawal of financial incentives for the General Practitioners after 12 months, some quality of life indicators (QIs) still improved, but there was a decrease in QI achievement rates after 18 months suggesting that the positive effects of time-limited financial incentives eventually diminish.

In summary, the articles on this Research Topic discussed some pertinent issues in the field of hypertension. The association of the intestinal barrier with hypertension, the association of hyperuricemia with left ventricular mass, and the AB blood group with preeclampsia was revealed. These findings will help clinicians identify the at-risk population and treat them proactively for the prevention and improved management of hypertension and related cardiovascular disease. The articles also discussed treating masked hypertension based on subphenotype in patients having associated metabolic syndrome and treating hypertension with nebivolol in sub-Saharan Africa. These findings could prove useful in hypertension treatment in specific populations.

The non-linear association of normal range systolic BP with all-cause mortality and cardiovascular death in older patients above 65 years of age and the non-linear association of SBP with in-hospital mortality of type A Aortic Dissection will help in determining an optimal BP target in these populations.

The suggested use of intermittent monitoring for assessing the rate of incident AF in hypertensives will help in assessing the association between blood pressure and incident AF. This will help in reaching the consensus on target BP for preventing new-onset AF. Lastly, a positive effects of time-limited financial incentive to general practitioners (GPs) was found on the proportion of patients achieving the recommended blood pressure target levels. After the withdrawal of financial incentives for the GPs after 12 months, some quality of life indicators (QIs) still improved, but there was decrease in QI achievement rates after 18 months, suggesting that 1 year might be too short to observe the full effect of such interventions and the positive effects of time-limited financial incentives eventually wane.

## Author contributions

All authors listed have made a substantial, direct, and intellectual contribution to the work and approved it for publication.

## Conflict of interest

The authors declare that the research was conducted in the absence of any commercial or financial relationships that could be construed as a potential conflict of interest.

## Publisher's note

All claims expressed in this article are solely those of the authors and do not necessarily represent those of their affiliated organizations, or those of the publisher, the editors and the reviewers. Any product that may be evaluated in this article, or claim that may be made by its manufacturer, is not guaranteed or endorsed by the publisher.
